# Prevalence and correlates of suicide attempts in juvenile bipolar and major depressive disorders

**DOI:** 10.1192/j.eurpsy.2025.1115

**Published:** 2025-08-26

**Authors:** L. Ciampoli, R. Abregú Crespo, E. Rodriguez Toscano, M. Ayora Rodriguez, R. Panadero Gómez, C. Ordás Díaz, A. Sánchez Cámara, L. Sevilla Cermeño, C. Llorente Sarabia, C. Moreno Ruiz

**Affiliations:** 1 Child Neuropsychiatry Unit, IRCCS Institute of Neurological Sciences of Bologna, Department of Medical and Surgical Sciences, University of Bologna, Bologna, Italy; 2 Department of Child and Adolescent Psychiatry, Institute of Psychiatry and Mental Health, Hospital General Universitario Gregorio Marañón, IiSGM, CIBERSAM, ISCIII, School of Medicine, Universidad Complutense, Madrid, Spain

## Abstract

**Introduction:**

Major depressive disorders (MDD) and bipolar disorders (BD) are strongly associated with suicide attempts in youth. However, little is known regarding the differences in prevalence and clinical-related features of suicide attempts between these two disorders.

**Objectives:**

The study aimed to examine the prevalence of suicide attempts and related factors in children and adolescents with major mood disorders and compare clinical features between BD and MDD patients who attempted suicide.

**Methods:**

A cross-sectional study was conducted at the Child and Adolescent Psychiatry Department of the Institute of Psychiatry and Mental Health Gregorio Marañón. Patients with diagnosis of major mood disorder were included. All patients were evaluated with K-SADS, CGI (Clinical Global Impression), HDRS (Hamilton Depression Rating scale), YMRS (Young Mania Rating Scale), Conners Rating Scale and CGAS (Children’s Global Assessment Scale). Bivariate analysis was conducted to compare patients with and without suicide attempts and BD versus MDD attempters. Subsequently, a multivariate analysis was performed to assess the combined influence of suicide attempts related factors.

**Results:**

The sample included 145 patients (58 males, 87 females) aged 7 to 17 years (mean 15.15), with diagnosis of BD (55.2%) and MDD (44.8%). The prevalence of suicide attempts for MDD and BD were 58.5 % and 28.8 %, respectively (p< 0.001). 56,5 % of BD attempters reported medically serious suicide attempts versus 28.9% of MDD (p=0.03). 61 patients with suicide attempts were compared to 84 patients without suicide attempts. Suicide attempts were associated with female sex (p<0.001), internalizing disorders (p= 0.003), hopelessness (p=0.001), higher punctuation on HDRS score (p=0.03), suicidal ideation (p<0.001) and NSSI (p<0.001). In the multivariate analysis, it was found that female sex (p<0.001), suicidal ideation (p<0.001) and hopelessness (p=0.048) were independently related to suicide attempts. Comparing suicide attempters with BD versus MDD, BD patients showed higher impulsivity (p=0.04) and a more alcohol use proportion (p=0.04) than MDD patients.

**Conclusions:**

Female sex, suicidal ideation and hopelessness are strongly related to suicide attempts in youth with major mood disorder. The findings revealed some differences between BD and MDD for patients with suicide attempts and accentuate the need for early identification of BD and accurate differentiation between BD and MDD in children and adolescents, to treat them adequately and minimize suicidal behaviors.

**Disclosure of Interest:**

None Declared

